# Integrated palliative care for patients with advanced head and neck cancer: a retrospective Brazilian cohort study of its impact at the end of life

**DOI:** 10.31744/einstein_journal/2025AO1768

**Published:** 2025-09-08

**Authors:** Cecilia Eugenio, Claudio Roberto Cernea, Marco Aurelio Vamondes Kulcsar, Toshio Chiba, Flavio Carneiro Hojaij, Giovanna Mattos Ferreira, Yasmin Sá Cerqueira, Leandro Luongo Matos

**Affiliations:** 1 Universidade de São Paulo Surgical Sciences and Perioperative Medicine Faculdade de Medicina São Paulo SP Brazil Postgraduate Program in Anesthesiology, Surgical Sciences and Perioperative Medicine, Faculdade de Medicina, Universidade de São Paulo, São Paulo, SP, Brazil.; 2 Universidade de São Paulo Faculdade de Medicina São Paulo SP Brazil Faculdade de Medicina, Universidade de São Paulo, São Paulo, SP, Brazil.; 3 Universidade de São Paulo Instituto do Câncer do Estado de São Paulo "Octavio Frias de Oliveira" Faculdade de Medicina São Paulo SP Brazil Head and Neck Surgery, Instituto do Câncer do Estado de São Paulo "Octavio Frias de Oliveira", Hospital das Clínicas, Faculdade de Medicina, Universidade de São Paulo, São Paulo, SP, Brazil.; 4 Hospital Israelita Albert Einstein Faculdade Israelita de Ciências da Saúde Albert Einstein São Paulo SP Brazil Faculdade Israelita de Ciências da Saúde Albert Einstein, Hospital Israelita Albert Einstein, São Paulo, SP, Brazil.

**Keywords:** Head and neck neoplasms, Integrative palliative care, Supportive care, Terminal care, Survival, Death

## Abstract

**Objective:**

To evaluate whether integrated palliative care is associated with improved overall survival and better end-of-life care in patients with upper aerodigestive tract malignancies. Secondary outcomes included the proportion of deaths in the intensive care unit, number of emergency department visits, chemotherapy use in the last 30 days of life, and the need for palliative sedation.

**Methods:**

This retrospective, non-randomized cohort study included patients with upper aerodigestive tract malignancies who died during a five-year period. Patients were categorized based on whether they received outpatient follow-up by a specialized palliative care team. Healthcare and clinical outcomes were compared between the two groups.

**Results:**

Among the 1,313 consecutive patients, 292 (22.2%) received outpatient palliative care. These patients had a median overall survival 4.7 months longer than those not followed up by palliative care. They also received less chemotherapy in the last 30 days of life, had fewer emergency department visits, had fewer intensive care unit deaths, and required less palliative sedation.

**Conclusion:**

Outpatient follow-up by a specialized palliative care team was associated with longer survival and better end-of-life care. These findings highlight the potential benefits of integrating palliative care earlier in the treatment of patients with upper aerodigestive tract cancers.

## Highlights

■Outpatient palliative care follow-up increased median survival by 4.7 months in patients with advanced head and neck cancer.

■Patients receiving palliative care had fewer intensive care unit deaths, less chemotherapy in the final month of life, and a reduced need for palliative sedation.

■Integrated palliative care resulted in fewer emergency visits per month of follow-up, suggesting more efficient symptom management.

## Key Message

Integrated outpatient follow-up by a specialized palliative care team for patients with upper aerodigestive tract cancers improves end-of-life care quality and increases survival.

## In Brief

Outpatient palliative care follow-up in patients with advanced head and neck cancer was associated with a 4.7-month increase in median survival and improved end-of-life outcomes. These included fewer intensive care unit deaths, reduced chemotherapy use in the final month, and fewer emergency visits per month.

## Graphical abstract

**Figure:**
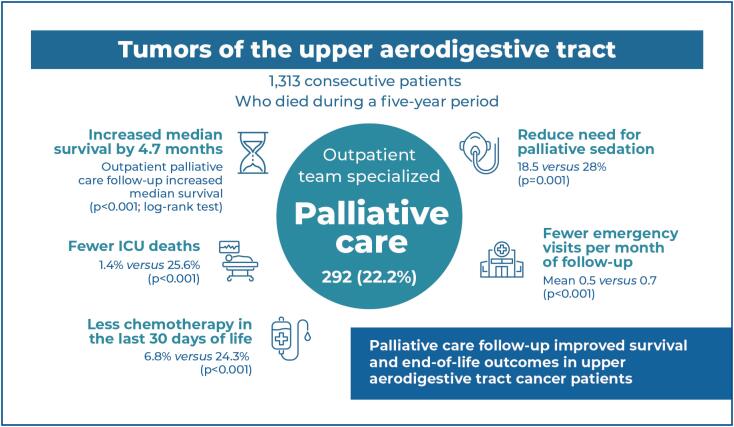

## INTRODUCTION

Most deaths in the United States occur in hospitals, often significantly affecting patients’ quality of life.^( 1 )^ In response, the number of palliative care units implemented in North American hospitals has been increasing.^( 2 )^ These teams have proven effective in managing symptoms for patients with advanced cancer, and they have also led to significant reductions in hospital expenditures.^( 3 )^ Specifically, hospital-based palliative care units have reduced costs by more than 60%, primarily by cutting intensive care unit (ICU)-related expenses.^( 4 )^ In Brazil, palliative care is recognized as a medical subspecialty, and head and neck surgery plays a key role in its development.

Malignant neoplasms of the upper aerodigestive tract account for approximately 5.3% of cancer-related deaths worldwide.^( 5 )^ Patients diagnosed with advanced-stage squamous cell carcinoma (SCC) in this region, particularly those with stage IV disease, have an estimated five-year survival rate of only 50%, even with curative treatment.^( 6 )^ These patients often experience a markedly diminished quality of life due to both the disease and the adverse effects of treatment. In this context, early integration of palliative care may not only improve symptom control but also help ensure more rational and appropriate use of healthcare resources.

Several well-established studies have demonstrated that early palliative care for patients with advanced solid tumors improves quality of life and may even extend survival.^( 7 - 9 )^ While most of these studies have focused on patients with non-small cell lung cancer, the biological behavior, prognosis, and clinical course of these tumors are comparable to those of upper aerodigestive tract SCCs. However, to date, no Brazilian study has specifically examined the role of early outpatient palliative care for this patient population.

## OBJECTIVE

This study aimed to evaluate the impact of early outpatient follow-up by a specialized palliative care team during the final phase of life of patients with upper aerodigestive tract malignancies. The outcomes measured included the location of death (particularly intensive care unit admissions), use of sedation in the last 24 hours of life, chemotherapy administration within 30 days of death, number of emergency room visits, and overall survival.

## METHODS

### Ethical considerations

This study was approved by the Institutional Ethics Committee of the *Hospital das Clínicas, Faculdade de Medicina, Universidade de São Paulo* (CAAE: 29898520.5.0000.0068; #3,974,781), in accordance with the Declaration of Helsinki. Since it involved only deceased patients, informed consent was waived.

### Study design and sample profile

This retrospective cohort study included consecutive patients treated at the *Instituto do Câncer do Estado de São Paulo do Hospital das Clínicas da Faculdade de Medicina da Universidade de São Paulo* (ICESP HCFMUSP), who died from malignant neoplasms of the upper aerodigestive tract between January 1, 2015, and December 31, 2019. Inclusion criteria were: age over 18 years; confirmed diagnosis of upper aerodigestive tract malignancy, defined by the following ICD-10 codes: oral cavity (C02, C03, C04, C05.0, C06), oropharynx (C01, C05.1, C05.2, C09, C10), nasopharynx (C11), hypopharynx (C12, C13), and larynx (C32); treatment at the study institution; and documented date of death within the defined period. Tumors of the lip were excluded due to challenges in classifying them as mucosal vs. cutaneous in origin. Skin cancers and labial lesions limited to the vermilion border or external lip surface were also excluded.

### Study groups

The main exposure of interest was outpatient follow-up by a specialized palliative care team. Patients were categorized into two groups: those who underwent regular outpatient palliative care follow-up and those who did not, although all patients received standard cancer treatment and supportive care. Referral to the outpatient palliative care clinic were not protocolized and was based on the clinical judgment of the attending physicians or the multidisciplinary team. As this was a retrospective observational study, no matching was performed between the groups.

### Palliative care model

The institutional palliative care team includes physicians trained in palliative medicine, nurses, psychologists, social workers, physical therapists, and speech-language pathologists. Outpatient follow-up typically begins with a medical consultation, after which patients have access to a full range of multidisciplinary support. This care is available to all patients treated at the institution, regardless of formal enrollment in the outpatient palliative care program.

### Analytical strategy

The primary outcome was overall survival, defined as the time from the initiation of cancer treatment to death. Secondary outcomes included death in occurring the ICU, chemotherapy administration within 30 days of death, emergency department visits during the final month of life, and use of palliative sedation in the last 24 hours of life. These variables were selected to as indicators of the quality of end-of-life care.

### Statistical analysis

Quantitative variables were summarized using the mean, median, standard deviation, and range (minimum and maximum). Categorical variables were expressed as absolute and relative frequencies. Normality of the quantitative variables was assessed using the Kolmogorov-Smirnov test. Between-group comparisons of continuous variables were performed using Student's *t* -test, and categorical variables were compared using the χ^2^ test. Survival analysis was conducted using the Kaplan-Meier method with log-rank tests to compare curves. Cox univariate regression was used to estimate risk, expressed as hazard ratios (HRs) with 95% confidence intervals (95CIs). All statistical analyses were performed using SPSS^®^ version 28.0 (IBM^®^ Inc., Armonk, NY, USA), with a two-tailed significance level set at p<0.05.

## RESULTS

### Sample profile

Between January 2015 and December 2019, 1,313 patients treated with ICESP died from malignant neoplasms of the upper aerodigestive tract. Of these, 191 (14.5%) were female and 1,122 (85.5%) were male. The most frequent site of death was the hospital ward (483 cases, 36.8%), followed by external settings (413 cases, 31.5%), the ICU (265 cases, 20.2%), the emergency department (133 cases, 10.1%), and the outpatient clinic (19 cases, 1.4%).

The most common primary tumor sites were the oropharynx (426 cases, 32.8%), oral cavity (373 cases, 28.7%), larynx (320 cases, 24.7%), hypopharynx (132 cases, 10.2%), and nasopharynx (47 cases, 3.6%). Additional epidemiological variables including education level, marital status, and self-reported race/skin color are presented in table 1 .

**Table 1:** Comparison of demographic characteristics between patients followed by the specialized palliative care team and those who did not receive this intervention

Variables		Total (n=1,313) n (%)	Palliative care outpatient clinic (n=292) n (%)	No palliative care (n=1,021) n (%)	p value (χ^2^ test)
Sex					
	Female	191 (14.5)	54 (18.5)	137 (13.4)	0.030
	Male	1,122 (85.5)	238 (81.5)	884 (86.6)	
Place of death					
	Outpatient clinic	19 (1.4)	2 (0.7)	17 (1.7)	<0.001
	Emergency room	133 (10.1)	46 (15.8)	87 (8.5)	
	Wards	483 (36.8)	132 (45.2)	351 (34.4)	
	External	413 (31.5)	108 (37)	305 (29.9)	
	Intensive care unit	265 (20.2)	4 (1.4)	261 (25.6)	
Primary tumor site					
	Oral cavity	373 (28.7)	77 (26.4)	294 (29.3)	0.453
	Oropharynx	426 (32.8)	99 (33.9)	327 (32.6)	
	Nasopharynx	47 (3.6)	13 (4.5)	34 (3.4)	
	Hypopharynx	132 (10.2)	36 (12.3)	96 (9.5)	
	Larynx	320 (24.7)	67 (22.9)	253 (25.2)	
Education level					
	Illiterate	41 (8.3)	12 (12.0)	29 (7.4)	0.069
	Incomplete elementary education	177 (36.0)	32 (32.0)	145 (36.9)	
	Complete elementary education	195 (39.6)	44 (44.0)	151 (38.5)	
	Incomplete high school	14 (2.9)	0 (0.0)	14 (3.6)	
	Complete high school	48 (9.7)	7 (7.0)	41 (10.5)	
	Incomplete university degree	3 (0.6)	2 (2.0)	1 (0.3)	
	Complete university degree	14 (2.9)	3 (3.0)	11 (2.8)	
Marital status					
	Married	584 (46.9)	124 (43.9)	460 (47.8)	0.785
	Divorced	162 (13.1)	36 (12.8)	126 (13.1)	
	Single	368 (29.6)	91 (32.3)	277 (28.8)	
	Consensual union	21 (1.7)	5 (1.8)	16 (1.7)	
	Widower	108 (8.7)	26 (9.2)	82 (8.5)	
Race/Skin color					
	Yellow or indigenous	6 (0.5)	1 (0.4)	5 (0.6)	0.812
	White	803 (69.1)	190 (69.1)	613 (69.0)	
	Black	71 (6.1)	14 (5.1)	57 (6.4)	
	Brown	283 (24.3)	70 (25.4)	213 (24.0)	

### Study groups

Out of the total 1,313 patients, 292 (22.2%) received outpatient follow-up by a specialized palliative care team. The mean duration between the first palliative care consultation and death was 133.3 days, with patients typically referred for follow-up 20 months after admission to the institution (median: 13 months).

Female patients were more likely to be followed by the palliative care team than male patients (p=0.030). There was also a trend suggesting that illiterate or poorly literate patients were more often referred to the palliative care team (p=0.069). However, no statistically significant differences were observed between the groups with respect to age (p=0.359), color (p=0.812), primary tumor site (p=0.453), marital status (p=0.485), or follow-up by the head and neck surgery team (p=0.292).

Patients without palliative care follow-up were significantly more likely to die in the ICU (261 cases, 25.6% *versus* 4 cases, 1.4%; p<0.001) and receive palliative sedation in the last 24 hours of life (286 cases, 28.0% *versus* 54 cases, 18.5%; p=0.001). Among patients who died in hospital wards (483 cases), sedation was administered in 83 of 351 cases (23.6%) for those not followed by the palliative care team, compared to 41 of 132 cases (31.1%) for those who were followed up (p=0.096).

Patients who received palliative care were less likely to undergo chemotherapy in the last 30 days of life (20 cases, 6.8% *versus* 248 cases, 24.3%; p<0.001) and had a longer interval between their last chemotherapy and death (mean: 194.3 ± 203.4 days *versus* 158.8 ± 321.7 days; p=0.030). Additionally, while patients followed by the palliative care team had more emergency room visits in total (mean: 6.3 ± 4.3 *versus* 4.5 ± 3.4 visits; p<0.001), the number of visits per month of follow-up was significantly lower (0.5 ± 0.6 *versus* 0.7 ± 1.1 visits/month; p<0.001). All these comparisons are summarized in table 2 .

**Table 2:** Comparison of treatment-related data between patients followed by a specialized palliative care team and those who did not receive this intervention

Variables	Palliative care outpatient clinic (n=292)	No palliative care (n=1,021)	p value *
Chemotherapy in the last 30 days of life, n (%)				
	Yes	20 (6.8)	248 (24.3)	<0.001
	No	272 (93.2)	773 (75.7)	
Death in the intensive care unit, n (%)				
	Yes	4 (1.4)	261 (25.6)	<0.001
	No	288 (98.6)	760 (74.4)	
Head and neck surgery patients, n (%)				
	Yes	249 (85.3)	844 (82.7)	0.292
	No	43 (14.7)	177 (17.3)	
Sedation in the last 24 hours of life, n (%)				
	Yes	54 (18.5)	286 (28)	0.001
	No	238 (81.5)	735 (72)	
Age				
	Number	292	1.021	0.359
	Minimum	21	18	
	Maximum	93	93	
	Mean	60.8	61.0	
	SD	10.3	10.6	
Emergency room admissions				
	Number	285	952	<0.001
	Minimum	1	1	
	Maximum	23	27	
	Mean	6.3	4.5	
	SD	4.3	3.4	
Emergency room visits per month of follow-up				
	Number	285	952	<0.001
	Minimum	0	0	
	Maximum	6.3	14	
	Mean	0.5	0.7	
	SD	0.6	1.1	
Time between death and last chemotherapy (days)				
	Number	209	656	0.030
	Minimum	1	0	
	Maximum	1.525	2.229	
	Mean	194.3	158.8	
	SD	203.4	321.7	

* p value obtained by the χ^2^ test for comparisons between groups and Student's *t* test for the comparison of means.

SD: standard deviation.

### Survival analysis

**Figure 1:**
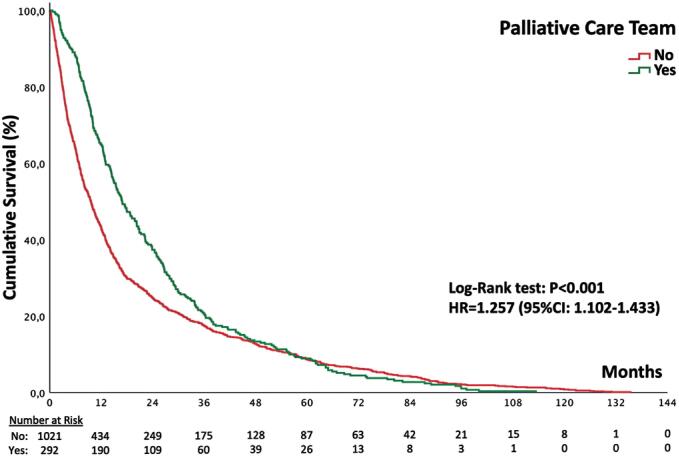
Kaplan-Meier curve showing that patients followed by the palliative care team had longer survival from the date of their first consultation at the institution until death (p<0.001; log-rank test). Median survival was 24.3 months in the group followed by the palliative care team, compared to 19.6 months in those who did not receive this follow-up-a difference of 4.7 months

Survival was significantly longer in patients followed by the palliative care team (p<0.001; log-rank test). As the biopsy date was unavailable, the first institutional consultation was used as the starting point for survival analysis. The median survival was 24.3 months in the palliative care group compared to 19.6 months in the control group, an increase of 4.7 months. This difference was particularly evident during the first 36 months of follow-up ( Figure 1 ). Absence of palliative care follow-up was associated with a higher risk of death during follow-up (HR = 1.257; 95%CI = 1.102-1.433; Cox regression).

## DISCUSSION

This study suggests that outpatient follow-up by a specialized palliative care team in patients with upper aerodigestive tract malignancies is associated with higher-quality end-of-life care and a modest, but clinically relevant, increase in survival. Patients who received palliative care had significantly fewer ICU deaths, reduced chemotherapy use in the final 30 days of life, lower need for palliative sedation, and fewer emergency department visits when adjusted for follow-up duration. Notably, median survival was 4.7 months longer in the palliative care group, a difference comparable to the gains observed with palliative systemic therapies.

Among the 1,313 patients analyzed, 292 (22.2%) had access to an outpatient palliative care clinic. This proportion remains low, even though the service was available at the institution. Similar global disparities have been documented. For instance, Boëthius et al. reported that only 14.2% of patients accessed dedicated palliative care units,^( 10 )^ and the World Health Organization (WHO) estimates that merely 14% of those in need receive such care.^( 11 )^ The American Society of Clinical Oncology recommends initiating palliative care within 8 weeks of an advanced cancer diagnosis.^( 8 )^ In our cohort, the median interval from institutional admission to the first palliative care visit was 13 months, indicating underutilization of early referral strategies. Although initial staging data was unavailable, prior Brazilian studies suggest that over 80% of upper aerodigestive tract cancers are diagnosed in advanced stages,^( 12 , 13 )^ supporting the need for earlier palliative care referral.

Sex-based differences were observed in referral patterns, with women being more frequently referred to the palliative care team. This aligns with findings by Hoerger et al.,^( 14 )^ who demonstrated a greater preference for palliative care among women. However, this contrasts with Civantos et al.,^( 15 )^ who reported no gender differences in referral patterns.

Patients in the palliative care group were significantly less likely to die in the ICU (1.4% *versus* 25.6%). This finding is consistent with studies conducted in other populations. Kuo et al.^( 16 )^ reported a 23.2% ICU death rate among patients with head and neck cancer in the U.S., while Ethunandan et al.^( 17 )^ found no ICU deaths when a palliative care nurse was included in the multidisciplinary team. Furthermore, palliative care was associated with reduced chemotherapy use in the last 30 days of life, which may reflect avoidance of overly aggressive treatments. Prigerson et al.^( 18 )^ demonstrated that such treatments can negatively impact the quality of life in patients with a good performance status. In our cohort, the mean interval between the last chemotherapy session and death was longer in the palliative care group, further supporting the role of early palliative care in promoting less aggressive care near the end of life.^( 19 , 20 )^

Palliative sedation was more common among patients without palliative care follow-up (28.0% *versus* 18.5%), which may indicate delayed symptom management. International studies have shown wide variability in the prevalence of sedation, ranging from 2.2% in Colombian palliative care units^( 21 )^ to 27.8% in Dutch hospices,^( 22 )^ depending on the practice setting and integration of palliative services.^( 23 )^

Although the absolute number of emergency visits was higher in the palliative care group, this difference reversed when adjusted for the significantly longer survival time. This reinforces the need to contextualize healthcare utilization over time. Previous studies have also highlighted high emergency department use near the end of life,^( 24 - 26 )^ but few adjusted for follow-up duration, which is a critical distinction in our analysis.

The observed survival benefit, despite the late average timing of referral (4.4 months before death), is noteworthy. Considering the large standard deviation (6.6 months) and the wide range in referral times (from 1 day to 56 months), earlier referrals may yield even greater benefits. It is also possible that patients with poorer performance status were preferentially referred, given that palliative care in Brazil is often associated with end-of-life care. If this is the case, the survival advantage observed in this potentially fragile population further supports the need for early palliative care integration.

This study has several limitations that should be considered when interpreting the results. First, as a retrospective observational study, it is not possible to establish causal relationships between the intervention and outcomes. The absence of randomization may have introduced selection bias, particularly if patients with poorer prognosis, more advanced disease, or greater comorbidity burdens were more likely to be referred—or not referred—to the outpatient palliative care team. Since group allocation was based on whether patients received outpatient palliative care follow-up, these differences could have influenced outcomes independent of the intervention itself.

Second, the hospital database used for analysis did not include information on disease stage or comorbidities, which precluded matching or statistical adjustment for prognostic variables, such as tumor burden, performance status ( *e.g* ., ECOG or Karnofsky scores), or systemic conditions. As a result, comparability between the groups may have been compromised. However, prior national data indicate that over 80% of upper aerodigestive tract cancers in Brazil are diagnosed at advanced stages,^( 12 , 13 )^ and that the entire study population included only deceased patients, further supporting the assumption that both groups comprised severely ill individuals with advanced disease.

Third, the study design introduces the possibility of time-related bias. As group categorization was based on whether a patient received outpatient palliative care, patients who died shortly after diagnosis or recurrence may not have survived long enough to be referred. Although these patients were not excluded from the study, this may have introduced a survival bias, inadvertently selecting for patients with longer life expectancy in both groups. While this does not invalidate the observed differences in quality-of-care indicators, it limits the interpretation of survival advantage.

Lastly, the study was conducted at a single tertiary cancer center—ICESP, which treats approximately 25% of all malignant neoplasms in the State of São Paulo. While this enhances the sample size and data reliability, the findings may not be generalizable to other institutions with different referral structures or practices. Additionally, although some patients in the "no outpatient palliative care" group may have received palliative support during hospital admissions, this was not systematically documented. If inpatient care was provided, it could have reduced the observed differences between the groups, reinforcing the potential benefit of integrating outpatient palliative care.

## CONCLUSION

This retrospective study suggests that outpatient follow-upby a specialized palliative care team for patients with upper aerodigestive tract cancer can significantly improve end-of-life care. Key outcomes include fewer intensive care unit deaths, reduced chemotherapy use in the last month of life, lower need for palliative sedation, and fewer emergency visits relative to follow-up time. Furthermore, patients who received palliative care had a median survival increase of 4.7 months. These findings underscore the potential value of early palliative care integration for this patient population.

DATA AVAILABILITY STATEMENT

Data supporting the findings of this study are available from the corresponding author upon request.

## References

[1] Morrison RS, Penrod JD, Cassel JB, Caust-Ellenbogen M, Litke A, Spragens L, Meier DE, Palliative Care Leadership Centers’ Outcomes Group (2008). Cost savings associated with US hospital palliative care consultation programs. Arch Intern Med.

[2] Penrod JD, Deb P, Luhrs C, Dellenbaugh C, Zhu CW, Hochman T (2006). Cost and utilization outcomes of patients receiving hospital-based palliative care consultation. J Palliat Med.

[3] de Santiago A, Portela MA, Ramos L, Larumbe A, Urdiroz J, Martínez M (2012). A new palliative care consultation team at the oncology department of a university hospital: an assessment of initial efficiency and effectiveness. Support Care Cancer.

[4] Hanson LC, Usher B, Spragens L, Bernard S (2008). Clinical and economic impact of palliative care consultation. J Pain Symptom Manage.

[5] Aupérin A (2020). Epidemiology of head and neck cancers: an update. Curr Opin Oncol.

[6] Döbrossy L (2005). Epidemiology of head and neck cancer: magnitude of the problem. Cancer Metastasis Rev.

[7] Temel JS, Greer JA, Muzikansky A, Gallagher ER, Admane S, Jackson VA (2010). Early palliative care for patients with metastatic non-small-cell lung cancer. N Engl J Med.

[8] Ferrell BR, Temel JS, Temin S, Alesi ER, Balboni TA, Basch EM (2017). Integration of palliative care into standard oncology care: american society of clinical oncology clinical practice guideline update. J Clin Oncol.

[9] Zimmermann C, Swami N, Krzyzanowska M, Hannon B, Leighl N, Oza A (2014). Early palliative care for patients with advanced cancer: a cluster-randomised controlled trial. Lancet.

[10] Boëthius H, Saarto T, Laurell G, Farnebo L, Mäkitie AA (2021). A Nordic survey of the management of palliative care in patients with head and neck cancer. Eur Arch Oto-Rhino-Laryngol.

[11] World Health Organization (WHO) (2022). Palliative care.

[12] Matos LL, Dedivitis RA, Kulcsar MA, De Mello ES, Alves VA, Cernea CR (2017). External validation of the AJCC cancer staging manual, 8th edition, in an independent cohort of oral cancer patients. Oral Oncol.

[13] Toledo LM, de Oliveira AS, Pinheiro RA, Leite AK, de Mello ES, Moyses RA (2021). Implication of the new AJCC pT classification of SCC of the Lip comparing with other oral subsites. Laryngoscope.

[14] Hoerger M, Perry LM, Gramling R, Epstein RM, Duberstein PR (2017). Does educating patients about the Early Palliative Care Study increase preferences for outpatient palliative cancer care? Findings from Project EMPOWER. Health Psychol.

[15] Civantos AM, Prasad A, Carey RM, Bur AM, Mady LJ, Brody RM (2021). Palliative care in metastatic head and neck cancer. Head Neck.

[16] Kuo TL, Lin CH, Jiang RS, Yen TT, Wang CC, Liang KL (2017). End-of-life care for head and neck cancer patients: a population-based study. Support Care Cancer.

[17] Ethunandan M, Rennie A, Hoffman G, Morey PJ, Brennan PA (2005). Quality of dying in head and neck cancer patients: a retrospective analysis of potential indicators of care. Oral Surg Oral Med Oral Pathol Oral Radiol Endod.

[18] Prigerson HG, Bao Y, Shah MA, Paulk ME, LeBlanc TW, Schneider BJ (2015). Chemotherapy use, performance status, and quality of life at the end of life. JAMA Oncol.

[19] Vukkadala N, Fardeen T, Ramchandran K, Divi V (2021). End-of-life practice patterns in head and neck cancer. Laryngoscope.

[20] Wu CC, Hsu TW, Chang CM, Lee CH, Huang CY, Lee CC (2016). Palliative chemotherapy affects aggressiveness of end-of-life care. Oncologist.

[21] Parra Palacio S, Giraldo Hoyos CE, Arias Rodríguez C, Mejía Arrieta D, Vargas Gómez JJ, Krikorian A (2018). Palliative sedation in advanced cancer patients hospitalized in a specialized palliative care unit. Support Care Cancer.

[22] van Deijck RH, Hasselaar JG, Verhagen SC, Vissers KC, Koopmans RT (2016). Patient-related determinants of the administration of continuous palliative sedation in hospices and palliative care units: a prospective, multicenter, observational study. J Pain Symptom Manage.

[23] Caraceni A, Speranza R, Spoldi E, Ambroset CS, Canestrari S, Marinari M, Marzi AM, Orsi L, Piva L, Rocchi M, Valenti D, Zeppetella G, Zucco F, Raimondi A, Matos LV, Brunelli C, Italian Society of Palliative Care Study Group on Palliative Sedation in Adult Cancer Patients (2018). Palliative Sedation in Terminal Cancer Patients Admitted to Hospice or Home Care Programs: Does the setting matter? Results from a national multicenter observational study. J Pain Symptom Manage.

[24] Heinonen T, Loimu V, Saarilahti K, Saarto T, Mäkitie A (2018). End-of-life care pathway of head and neck cancer patients: single-institution experience. Eur Arch Oto-Rhino-Laryngol.

[25] Chang TS, Su YC, Lee CC (2015). Determinants for aggressive end-of-life care for oral cancer patients: a population-based study in an Asian country. Medicine (Baltimore).

[26] Ullgren H, Kirkpatrick L, Kilpeläinen S, Sharp L (2017). Working in silos? - Head & Neck cancer patients during and after treatment with or without early palliative care referral. Eur J Oncol Nurs.

